# Sheep Dips

**Published:** 1891-06

**Authors:** 


### SHEEP DIPS.

For the second time, our inspectors of scab areas have sent in annual reports upon the different sheep dips used in their several districts.

From these reports—twenty-five in all—we gather the following most useful information in regard both to the relative efficacy of the dips according to the experience of sheep-owners, and the extent to which such dips are respectively used—and it is in the latter degree that they are tabulated as follows :—

1. LIME AND SULPHUR. No less than twenty-four areas concur in the opinion that this preparation ranks first in the generality of its use and as a killer of scab. Its cost, however, is slightly greater than that of some of the other dips. The ingredients and mode of preparation of this dip are as follows, viz :—Lime (slaked) and sulphur, 18 lbs. of each (minimum strength), to 100 gallons of water. The lime is at first slowly slaked in the usual way, then the rest of the water and the sulphur added. This mixture, when boiled for about twenty minutes, is to be well strained, and when used for dipping brought to a temperature of about 100° Fahrenheit.

2. COOPER'S DIP, comes next in order of merit and in extent of use, and in most districts is considered second only to "lime and sulphur," twenty-one areas reporting very favorably as to its efficacy.

3. TOBACCO, eighteen areas report favorably of the effectiveness and extensive use of tobacco dip, the mode of preparing which is as follows, viz :—Tobacco extract, 25 lbs. (minimum strength) of good, sound, fermented tobacco to 100 gallons of water. The mixture should be prepared by steeping the tobacco in cold water for not less than 24 hours, then, when about to use it, raise the water to the boiling point, immediately withdraw the fire and allow it to draw like tea for an hour, when it is fit for use. N. B.—The solution should be thoroughly strained by pressure from the leaves.

4. LITTLE'S DIP.—The majority of reports concur in the statement that this dip, although not so much used as the dips beforementioned, is effective.

5. MCDOUGALL'S DIP.—And precisely the same thing is said in regard to this dip.

6. Quibell's Dip.—From four areas reports favorable have been sent in, but in other areas it appears to be so little used as to be practically almost unknown.

7 and 8. Glycerine and Hellier's Dip.—The same can be said concerning these dips, three areas reporting favorably about each, whilst in the remaining areas the dips are practically unknown.

9. Hayward's Dip.—Newly introduced. The two reports received are favorable, both adding that the dip is "very similar to Cooper's."

10. Tar Elixir.—Practically unknown in our scab areas.—*Agricultural Journal*, Cape Colony, Vol. III, No. 18.

### CLIPPING OF HORSES.

At a recent meeting of the *Société Centrale de Médecine Vétérinaire* the above subject was introduced by M. Lavalard, and an interesting discussion took place.

M. Lavalard said that the horses of the Omnibus Co., in Paris had not been clipped during the winter and the results were satisfactory. They escaped many of the ailments of previous winters, after which the clipped horses regained their flesh and new growth of hair with difficulty.

M. Leblanc said that his experience was that unclipped horses returned to the stables after work covered with sweat which did not dry during the night. This resulted in loss of appetite and strength both of which returned as soon as the animals were clipped.

M. Weber thought the accumulation of hair increased the number of respirations.

M. Laquerriere said that in his experience with cavalry horses in Algeria he found those which were clipped were always in better condition than the others. They should be clipped in October so that the hair would be slightly reproduced before the advent of extreme cold. He had once made an experiment to test the question. 100 horses, 20 from each squadron were clipped, 100 others were not clipped, all were in the same conditions as regards age, race etc. The 200 were weighed beforehand, and again at close of the winter. The clipped animals were found to weigh more than the others.

M. Aureggio was opposed to clipping. He said that the Government ordered all the horses in the army to be clipped in 1872, because they were in such a miserable condition after the war, and that the decree of 1883 prescribed clipping as an exceptional measure. Like the most of my confrères in the army I have never ordered the horses clipped except as a therapentic measure, and have never noticed an increase of the diseases of the chest or abdomen in consequence.

M. Laquerriere said that the care of the skin was much easier in clipped animals and that they showed more vigor and energy after the operation. Besides they are not so liable to be chafed by the harness or if chafed more easily healed, grooming is easier and more quickly done, whilst the function of the skin is maintained and cutaneous erruptions are prevented.

After the fatal Franco-German war the unfortunate horses which had taken part were found to be suffering from cutaneous erruptions. They were nearly all clipped but more to facilitate treatment rather than as a hygienic measure. In the years following clipping became general and the curry-comb suppressed. In many animals the operation was delayed until January and those suffered terribly. Those which had been clipped before the cold began were in good condition. I have made a series of careful experiments among the army horses and have come to the following conclusions :—

Clipping when performed at the proper time is an excellent hygienic operation for the horse. It increases its vigor and energy, diminishes cutaneous transpiration, favors the prompt drying of the skin when it is impregnated with sweat, in other words; it regulates the function of the skin, prevents the effects of cutaneous chill on the viscera, facilitates good grooming, and prevents chafing by the harness or facilitates treatment when chafing takes place.

M. Aureggio said that although clipping improved the appearance of the horse and made grooming easy it had serious disadvantages when not done under cover, in warm stables and with double rations of hay.

M. Decroix thought that the adoption of this operation as a general measure in the army would be unfortunate because all the regiments of cavalry are not under the same conditions of climate, etc.

M. Mattieu, a veterinarian of great experience, was of the opinion that if a horse be well nourished with good oats his hair will be short and brilliant and will never need be clipped, but if clipped under good hygienic conditions will profit by the operation. On the other hand if the supply of oats be small his skin will be dirty with long and dull hair and will not profit by clipping.

M. Aureggio said that clipped horses required an increased ration of oats, often an impossibility in the army, and for that reason an argument against clipping.

M. Menard said that circumstances should guide the hygienist or the economist in accepting or rejecting the operation. In the *Jardin d' acclimatation* some animals were never clipped while others were four or five times a year. These latter, Island Ponies, coming from a cold climate with thick and warm fur, like all animals from the North, would be absolutely unfit for service if never clipped even in mid-winter.

I believe, therefore, that each one should be guided by the requirements of each case and this is the only conclusion which can be drawn from the discussion.—*Receuil de Médecine Vétérinaire.*

